# Association of *TLR3* single nucleotide polymorphisms with susceptibility to HTLV-1 infection in Iranian asymptomatic blood donors

**DOI:** 10.1590/0037-8682-0026-2020

**Published:** 2020-06-22

**Authors:** Hossein Mehrabi Habibabadi, Masoud Parsania, Ali Akbar Pourfathollah, Setareh Haghighat, Zohreh Sharifi

**Affiliations:** 1 Department of Microbiology, Faculty of Advanced Science and Technology, Tehran Medical sciences, Islamic Azad University, Tehran, Iran.; 2 Department of Microbiology, Faculty of Medicine, Tehran Medical sciences, Islamic Azad University, Tehran, Iran.; 3 Department of Immunology, Faculty of Medical Sciences, Tarbiat Modares University, Tehran, Iran.; 4 Blood Transfusion Research Center, High Institute for Research and Education in Transfusion Medicine, Tehran, Iran

**Keywords:** HTLV-1, SNP, TLR3, Restriction *fragment length polymorphism*

## Abstract

**INTRODUCTION:**

The human T-lymphotropic virus type 1 (HTLV-1) has a single-stranded RNA genome and expresses specific proteins that have oncogenic potential. Approximately 15 to 20 million people worldwide have been infected by this virus. Changes in protein or gene expression are the effects of single nucleotide polymorphisms (SNPs) within the Toll-like receptor 3 (*TLR3*) gene. The function and efficacy of signal transduction also lead to modified immune responses. The present study aimed to investigate the association of SNPs within *TLR3* (rs3775291 and rs3775296) with susceptibility to HTLV-1 infection in Iranian asymptomatic blood donors.

**METHODS:**

This study was performed on 100 HTLV-1-infected asymptomatic blood donors and 118 healthy blood donors. Genomic DNA from all participants was purified and then amplified using specific PCR primers. SNPs within *TLR3* were evaluated using the restriction fragmentation length polymorphism technique, and the results were analyzed using SPSS software (version 22).

**RESULTS:**

The frequencies of the *TLR3* (rs3775296) CC, CA, AA genotypes were 70%, 24%, and 6% in the patient group, and 50.8%, 44.9%, and 4.2% in the control group, respectively. There was a significant difference in the frequency distribution of *TLR3* (rs3775296) genotypes and alleles, but not in the frequency distribution of *TLR3* (rs3775291) genotypes between the patient and control groups.

**CONCLUSIONS:**

The *TLR3* SNP rs3775296 was significantly associated with HTLV-1 infection and may be a protective factor against this viral infection.

## INTRODUCTION

Infection with human T-lymphotropic virus type 1 (HTLV-1) may cause disease. It is currently estimated that 15 to 20 million people worldwide are infected with this virus^1^ ^,^ ^2^. HTLV-1 infection is endemic in some parts of the world, including southern Japan, some Caribbean countries, sub-Saharan Africa, South America, Papua New Guinea, the islands of Melanesia and Solomon in Oceania, and northeastern Iran (Khorasan Province)^2^ ^,^ ^3^ ^,^ ^4^. 

The immune system plays a decisive role in determining the fate of infection by infectious agents. - (TLRs) are usually expressed on the cell surface and play significant roles in innate immune responses; 11 human TLR family members have been discovered. TLR3 is found in endosomal compartments where it recognizes retroviral double-stranded RNA and induces antiviral activities by producing inflammatory cytokines and type I interferons (IFNs).

Gene polymorphisms, SNPs, are common, appearing about 1% of the general population. They can lead to amino acid substitutions and altered gene promoter activity^5^ ^-^ ^8^, affecting gene expression, mRNA conformation and stability, or protein structure and function^9^. It has been suggested that polymorphisms in the *TLR3* promoter region may affect gene expression and cause transcriptional modulation of *TLR3* ^10^. TLR3, after binding to ligands, induces inflammatory cytokine production, triggering induction of type I IFNs through nuclear factor-κB, or interferon regulatory factor (IRF)-dependent signaling pathways. Amino acid substitution may be the result of the rs3775291 SNP. Mutations at this position may cause mRNA configuration changes and impair the function of TLR3 protein due to stringent purifying selection pressure related to the C allele. SNP rs3775296 is in the 5′-untranslated region (UTR) of *TLR3*. It has been suggested that several polymorphisms that alter amino acid residues in TLR3 can lead to altered protein structure and function. Therefore, they may cause downregulation of the expression of *TLR3* and reduce functional activity needed for appropriate signaling^11^.

Several SNPs located on *TLR3* (rs3775290, rs1879026, rs3775296, rs3775291, rs5743305 and rs13126816) have been selected as the targets of studies to assess the risk of viral infection and related diseases^12^ ^-^ ^16^. Since no studies have been performed on the possible association of *TLR3* polymorphisms with susceptibility to HTLV-1 infection, we aimed to investigate *TLR3* polymorphisms involving rs3775291 and rs3775296 in HTLV-1-infected individuals, in comparison to healthy individuals.

## METHODS

### Patients and samples

This case control study selected 100 blood samples from blood donors in Khorasan Razavi Province (which is an endemic area of HTLV-1 in Iran) who were found to be HTLV-1+ using ELISA (Diapro HTLV I-II Ab Kit, Milano, Italy) and a western blotting confirmatory test (MP kit, Singapore, Singapore) ). In addition, for control samples, a total of 118 blood donor specimens negative for HTLV-1 using ELISA (Diapro HTLV I-II Ab Kit, Milano, Italy) were selected. All participants were negative for Hepatitis B virus (HBV)), Hepatitis C virus (HCV), and *Human immunodeficiency virus* (HIV**)** tests. This study received ethics approval, with the number IR.IAU.PS.REC.1397.358 provided by the Ethics Committee of Islamic Azad University of Medical Sciences. Tehran, Iran. Also, all participants signed informed consent for entrance in the study.

### Genomic DNA extraction

We used blood genomic DNA extraction mini kits (FavorGen, Ping-Tung,Taiwan) for isolation of genomic DNA from the buffy-coats of all samples PingTnng-, based on the manufacturer’s instructions. Concentrations of genomic DNA were measured using a Nanodrop instrument (Denovix, *Wilmington*,).

### Identification of SNPs

Two SNPs, namely rs3775291 and rs3775296, within the *TLR3* gene were selected for this study^17^ ^,^ ^18^. Specific PCR primers were verified using the basic local alignment search tool (BLAST) at the NCBI site (https://www.ncbi.nlm.nih.gov/, Table 1). We prepared PCR amplification reagents in final volumes of 25 μL (each reaction) consisting of 2 × master mix (Ampliqon, Copenhagen, Denmark), 12.5 μL; forward primer (10 μM), 1 μL; primer reverse (10 μM), 1 μL; template (1-100 ng), 2.5 μL; and ddH_2_O, 8 μL. PCR amplification was performed using specific primers, and the temperature profile included an initial denaturation step at 95°C for 5 min, 35 cycles of 95°C for 45 s, 56°C for 45 s, 72°C for 45 s, and finally an extension step at 72°C for 10 min. Electrophoresis of PCR products was performed using 1.5% agarose gels, and DNA Safe stain (FavorGen -) was used. Next, the bands were verified by gel documentation (ATP, Tehran,Iran). After viewing the specific bands for all samples, overnight enzymatic digestion (Table 1) was performed on the PCR products. The products of enzymatic digestion were verified using 2.5% agarose gels and DNA Safe dye (FavorGenPingTnng-) at 100 volts, and bands were revealed by gel imaging (ATPTehran-). Through these means, restriction fragmentation length polymorphism (RFLP) genotyping was performed on all PCR products (Table 1). DNA sequencing was used to establish reference standards for analyzing genotypes using PCR-RFLP.

**TABLE 1:** Specifications of the study SNPs.

Gene	SNP	Method	Primer sequence (5′-3′)	Restriction enzyme	Genotype	Fragments size (bp)	Reference
*TLR3*	rs3775291	RFLP	GGCTAAAATGTTTGGAGCAC	HpyF3I*	TT	200	17
			TGAGATTTTATTCTTGGTTAGGCTGA		CT	31, 169, 200	
					CC	31,169	
	rs3775296	RFLP	GCATTTGAAAGCCATCTGCT	MboII*	AA	257, 17	18
			AAGTTGGCGGCTGGTAATCT		CA	279, 257, 17	
					CC	279	

*(Thermo Scientific, Lithuania).

### Statistical analysis

For statistical analysis, SPSS software v.22.0 (SPSS, Inc., Chicago, IL, USA) was used. Hardy-Weinberg equilibrium (HWE) testing was individually used for all SNPs using Pearson’s χ^2^ test. The χ^2^ test was used to compare the distribution of genotypes and frequencies of alleles between two groups. The level of statistical significance was set at 5%. Logistic regression analysis was applied to compute 95% confidence intervals (95% CI) and odds ratios (OR).

## RESULTS

Our study population included 75 men (75%) and 25 women (25%) in the case group, and 104 men (88.14%) and 14 women (11.86%) in the control group. The average ages of patients and controls were 38.55 ± 9.85 and 36.72 ± 9.93 years, respectively. Frequencies of the *TLR3*rs3775296 CC (wild type), CA (heterozygous), and AA (polymorphic homozygous) genotypes were 70%, 24%, and 6% in HTLV-1 patients, and 50.8%, 44.9%, and 4.2% in the control group, respectively. Moreover, frequency of the *TLR3* rs3775296 C allele was 82% and 73.3% in the HTLV-1 patient and control groups, respectively. There was a significant difference in the distribution of *TLR3* rs3775296 genotypes and frequencies of alleles between the patient and control groups (p = 0.002). A protective role of the genotypes CA with OR 0.388 and a 95% CI, of 0.215 to 0.702 and CA + AA with OR 0.443 and (95% CI, 0.253-0.776)], was observed against the disease (Table 2 and Table 3). In addition, frequencies of the *TLR3* rs3775291 CC (wild type), CT (heterozygous), and TT (polymorphic homozygous) genotypes were 52%, 43%, and 5% in the HTLV-1 patient group, and 46.6%, 47.5%, and 5.9% in the control group, respectively. The frequency of the *TLR3* rs3775291 C allele was also observed to be 70% in the HTLV-1 patient group and 70.3% in the control group. However, a statistically significant difference in the distribution of *TLR3* rs3775291 genotypes, as well as in allele frequencies between the patient and control groups was not detected (p = 0.4) (Table 2 and Table 3). In addition, the genotype distributions did not significantly deviate from Hardy-Weinberg expectations for both groups (p > 0.05) (rs3775296, 0.92; rs3775291, 0.07).

**TABLE 2:** Distribution of *TLR3* SNP genotypes in HTLV-infected cases and healthy controls.

SNP	Model	Genotype	Genotype frequencies; n (%)		p ^a^	OR (95% CI)
			HTLV-infected (100 cases)	Healthy (118 controls)		
rs3775296	***Codominant***	CC	70 (70.0%)	60 (50.8%)	_	ref
		CA	24 (24.0%)	53 (44.9%)	0.002	0.388 (0.215-0.702)
		AA	6 (6.0%)	5 (4.2%)	0.964	1.029 (0.299-3.540)
	***Dominant***	CC	70 (70.0%)	60 (50.8%)	_	ref
		CA + AA	30 (30.0%)	58 (49.2%)	0.004	0.443 (0.253-0.776)
	***Recessive***	CC + CA	94 (94%)	113 (95.8%)	_	ref
		AA	6 (6.0%)	5 (4.2%)	0.554	1.443 (0.427-4.876)
**rs3775291**	***Codominant***	CC	52 (52.0%)	55 (46.6%)	_	ref
		CT	43 (43.0%)	56 (47.5%)	0.458	0.812 (0.469-1.407)
		TT	5 (5.0%)	7 (5.9%)	0.649	0.755 (0.226-2.530)
	***Dominant***	CC	52 (52.0%)	55 (46.6%)	_	ref
		CT + TT	48 (48.0%)	63 (53.4%)	0.428	0.806 (0.473-1.374)
	***Recessive***	CC + CT	95 (95%)	111 (94.1%)	_	ref
		TT	5 (5.0%)	7 (5.9%)	0.764	0.835 (0.256-2.716)

**a:** P values were calculated using χ ^2^ test; **SNP:** single nuclear polymorphism, **OR:** odds ratio, **CI:** conﬁdence interval.

**TABLE 3:** Allele distribution of *TLR3* SNPs in HTLV-infected cases and healthy controls.

SNP	Allele	Allele frequencies; n (%)		P ^a^	OR (95% CI)
		HTLV-infected (100 cases)	Healthy (118 controls)		
rs3775296	C	164 (82.0%)	173 (73.3%)	_	ref
	A	36 (18.0%)	63 (26.7%)	0.031	0.603 (0.380-0.957)
rs3775291	C	147 (70.0%)	166 (70.3%)	_	ref
	T	63 (30.0%)	70 (29.7%)	0.938	1.016 (0.677-1.526)

**a:** P values were calculated using χ ^2^ test; **SNP:** single nuclear polymorphism, **OR:** odds ratio, **CI:** conﬁdence interval.

## DISCUSSION

Genetic alterations involving the TLR signaling pathway can cause susceptibility or resistance to several infectious diseases^19^ ^-^ ^21^. Given that TLR3 is capable of sensing dsRNA and stimulating the production of type I IFNs and inflammatory cytokines, it is clear that the receptor plays an important role in antiviral defense. In human, *TLR3-*promoter region has an important role in maintaining the integrity of the promoter and the specific responsive elements of the promoter to viral agents. Malignant mutations and changes involving leucine to phenylalanine substitution at TLR3 amino acid location 412 (Leu412Phe) are among the effects of the rs3775291 polymorphism in *TLR3* exon 4^22^. According to previous studies, this SNP does not affect the expression of TLR3, or its intracellular localization in vesicles, whereas it has negative effects on dsRNA binding ability and on cell surface expression of TLR3^23^ ^,^ ^24^. Moreover, it has been suggested that promoter polymorphisms, such as *TLR3* rs3775296, can influence gene expression in response to inflammatory cytokines and cause transcriptional modulation of *TLR3*. 

In the current study, no significant association was observed involving *TLR3* rs3775291 and susceptibility to HTLV-1 infection. However, there were significant differences in the distribution of *TLR3* rs3775296 genotypes and allele frequencies between the patient and control groups. It seems that these observations may indicate a protective factor to prevent HTLV-1 infection. The protective genotypes included CA [OR (95% CI)], 0.388 (0.215-0.702), and CA + AA [OR (95% CI)], 0.443 (0.253-0.776), against HTLV-1 infection. There have been no studies concerning the association of SNPs within *TLR3* (rs3775296 and rs3775291) with HTLV-1 infection in the Iranian population. To date, there are only reports regarding the association of SNPs within *TLR3* and other viruses such as HIV and HCV. In a study by Sironi et al. (2012), *TLR3* rs3775291 was genotyped in a group of Spanish HIV-seronegative individuals, despite their repeated exposure to HIV by i.v. injection drug use (IDU), whereas they had evidence of HCV-seropositivity. Significantly, the frequency of one homozygous rs3775291 allele in HIV-seronegative individuals was higher in comparison to controls. This research showed that a common *TLR3* allele confers an immunological advantage that can protect against HIV-1 infection and proposed the potential use of stimulation of TLR3 levels in HIV-1 immunotherapy^25^. Enhanced sensitivity of TLR3 to stimulation may be due to increased expression of TLR3 in cells harboring the rs3775291 allele. Additionally, there are multiple reports that have highlighted the link between expression of TLR and response to HIV infection^26^, as well as in other physiological or pathological conditions^27^ ^-^ ^29^. In a study by Fischer et al. (2018), the importance of the *TLR3* rs3775291 polymorphism was shown in the natural course of chronic HBV infection among patients. The polymorphic *TLR3* rs3775291 A allele is associated with decreased possibility of spontaneous clearance of Hepatitis B surface antigen (HBsAg) and Hepatitis B e-antigen (HBeAg) in serum, and an increased risk of developing chronic hepatitis B. Haplotype analysis revealed that the variant rs3775291A presents the lowest possibility of clearance of HBsAg in serum^30^. In a study by Guedes de Sá et al. (2015), the prevalence of two SNPs (rs3775291) in the *TLR3* gene was investigated in patients infected with HBV, HCV, and healthy controls. However, there were no significant differences in the frequencies of alleles, genotypes and haplotypes between the studied groups^16^. Zayed et al. (2017) in their study investigated possible associations involving genetic variations in TLR3 with HCV infection, and hepatic fibrosis in patients with chronic HCV in Egypt. Genotyping of *TLR3* rs3775296 was carried out on naïve chronic HCV-positive patients and on healthy controls using the PCR-RFLP technique. They reported that the frequency of polymorphic genotypes involving *TLR3* rs3775296 was not significantly different between HCV-positive patients and controls^18^. In a study by Motavaf et al. (2014), it was reported that patients with chronic HCV infection have lower levels of *TLR3* and *TLR7* expression^31^. 

Studzinska et al. (2016) investigated the association between *TLR3* rs3775291 and rs3775296 SNPs and viral infection in Cytomegalovirus (CMV)-positive children(Cytomegalovirus**)**. They reported an increase in the frequency of heterozygous *TLR3* rs3775291 genotypes in children with HCMV infection in comparison to uninfected controls. Being heterozygous at the rs3775291 SNP was related to increased risk of HCMV disease. In addition, individuals heterozygous at rs3775296 exhibited an increased relative risk of CMV infection, although this *association* was not statistically significant for multiple experiments after correction. 

Those authors suggested that polymorphism involving rs3775291 in *TLR3* could be a genetic risk factor for the development of CMV^32^.

Our results also revealed no significant association between the *TLR3* rs3775291 SNP and HTLV-1 infection. However, *TLR3* rs3775296 SNP was significantly associated with such infection and appears to be a protective factor against HTLV-1 infection. Finally, the possible influence of polymorphisms in terms of infection control mechanisms should be considered^18^ ^,^ ^20^.
